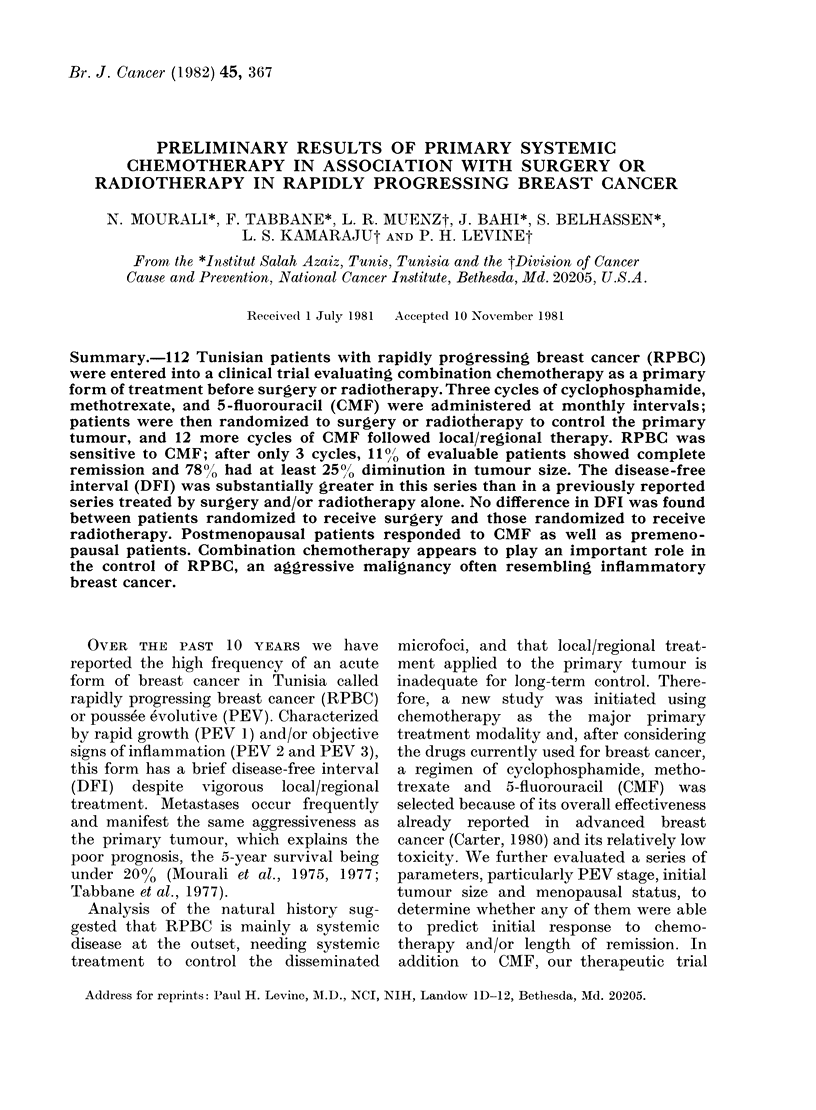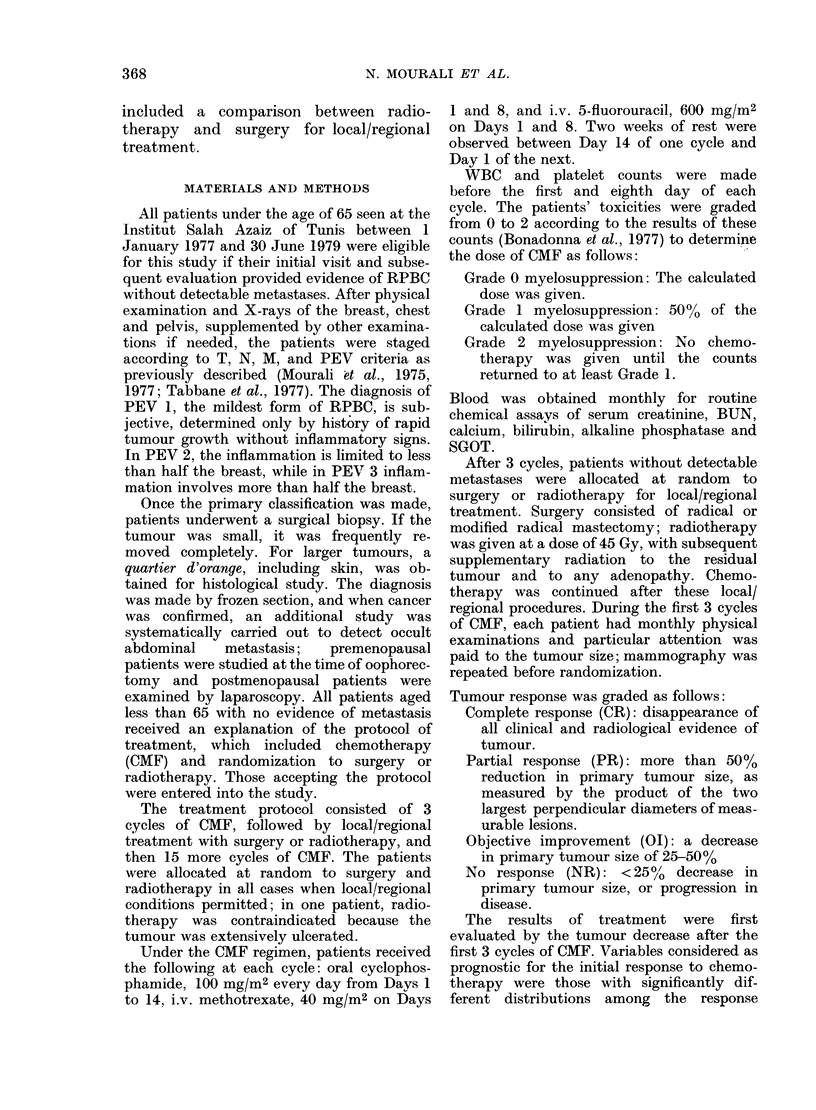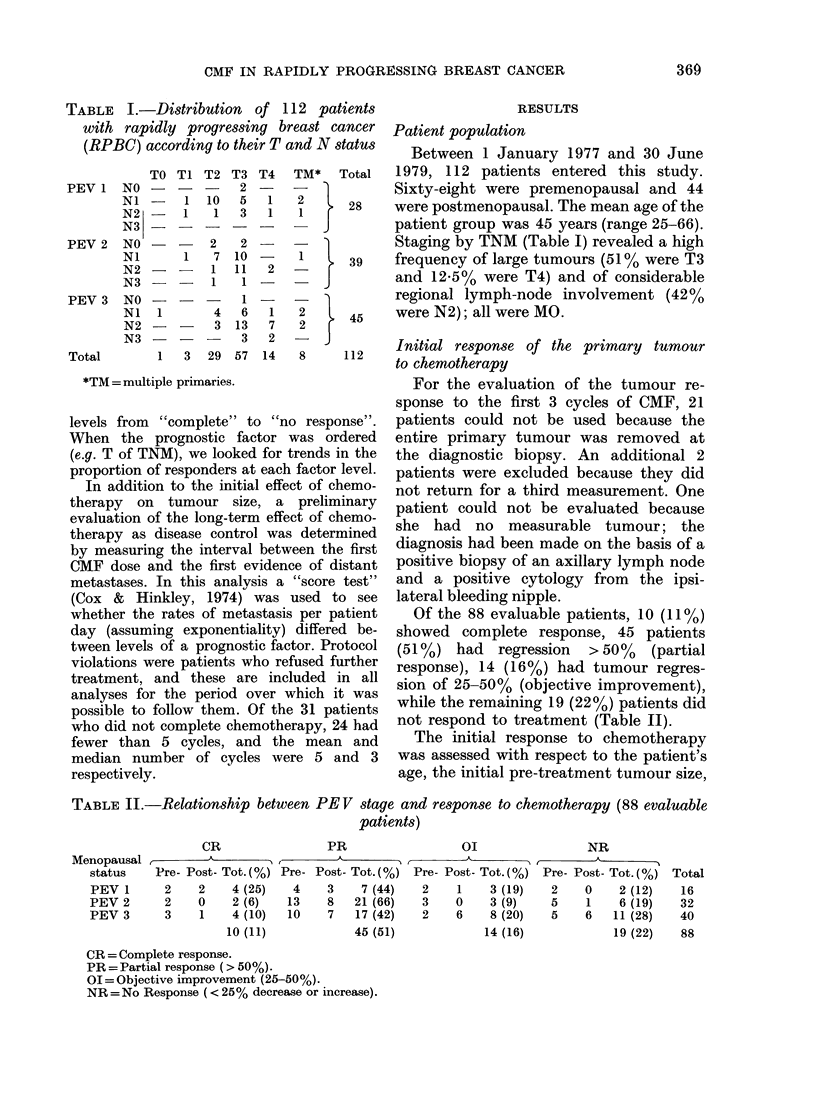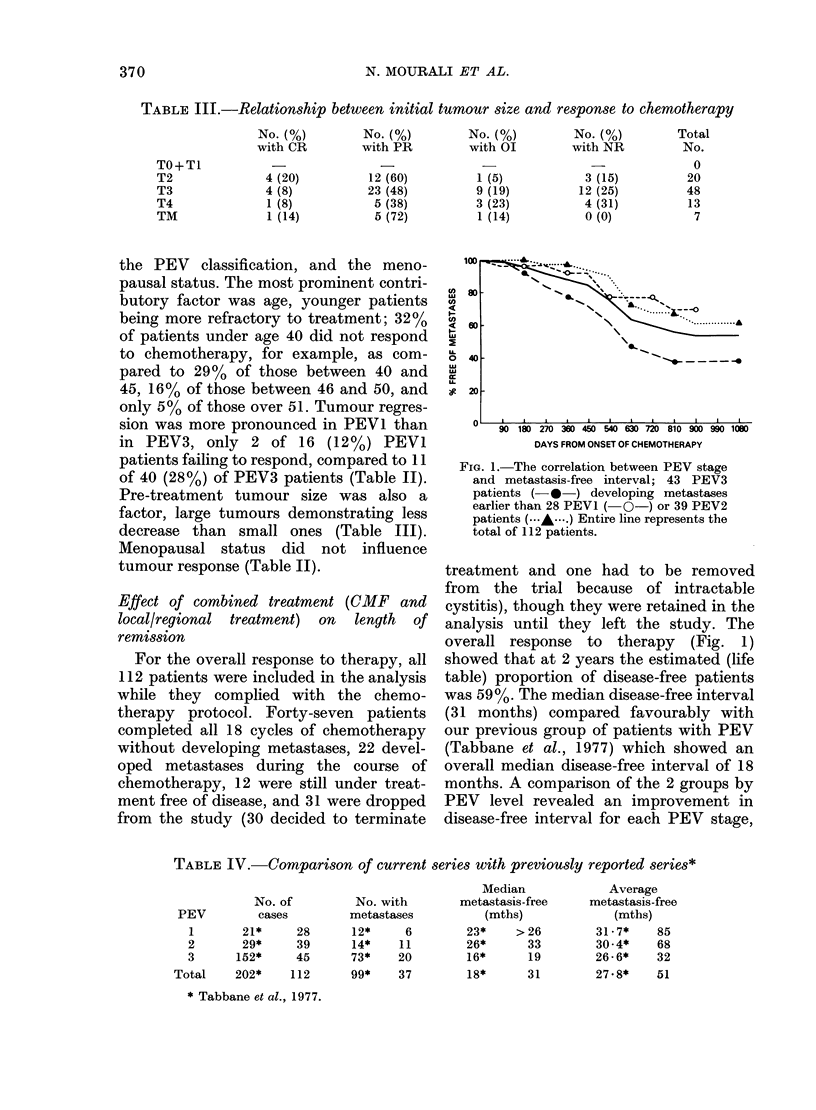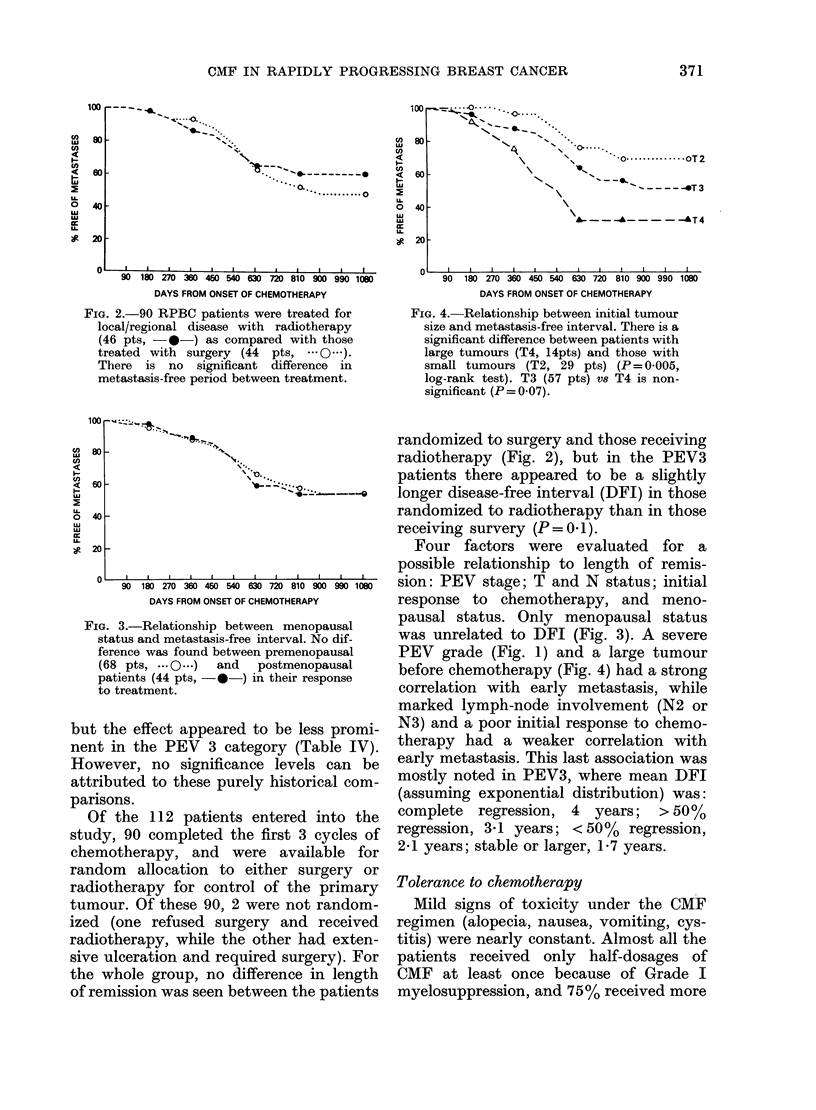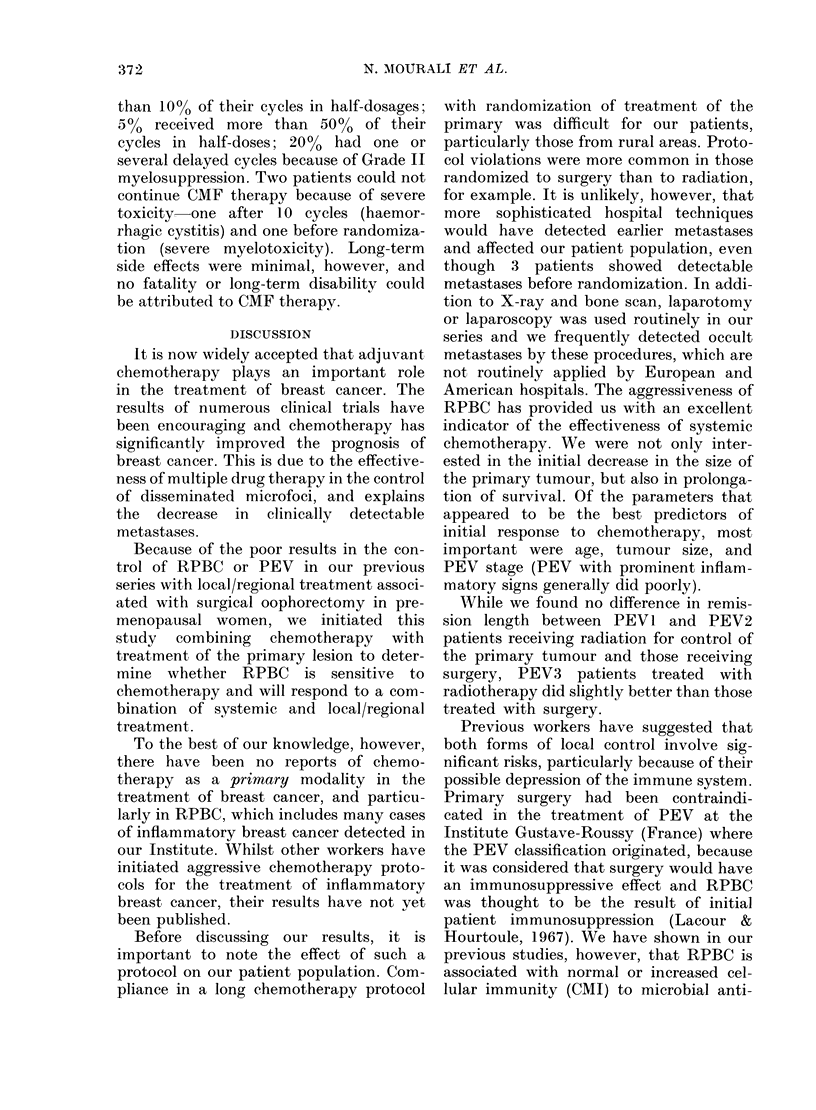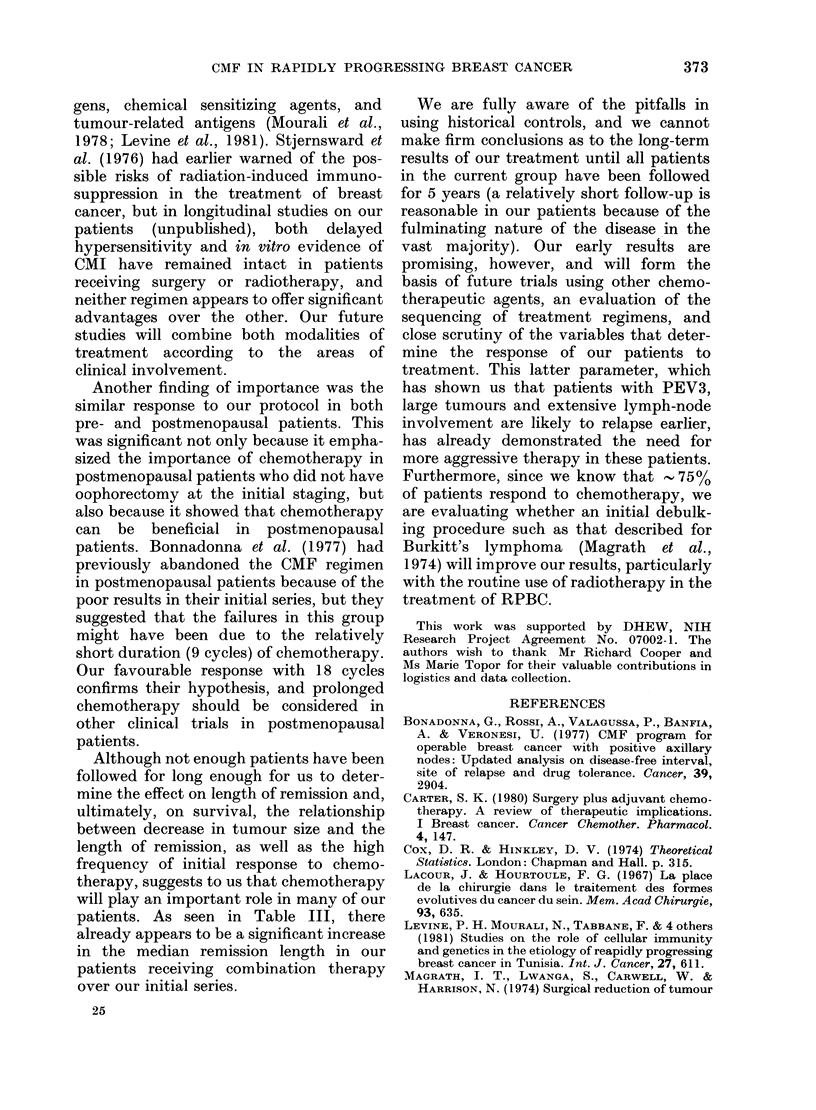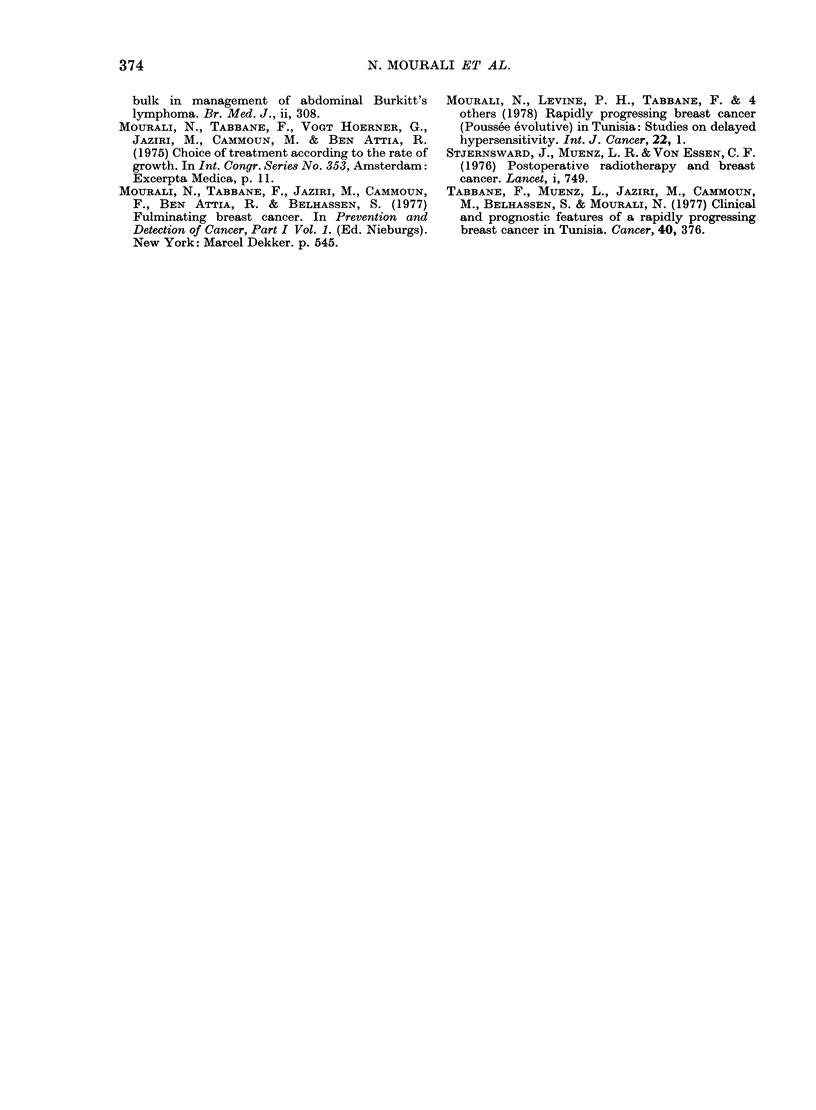# Preliminary results of primary systemic chemotherapy in association with surgery or radiotherapy in rapidly progressing breast cancer

**DOI:** 10.1038/bjc.1982.63

**Published:** 1982-03

**Authors:** N. Mourali, F. Tabbane, L. R. Muenz, J. Bahi, S. Belhassen, L. S. Kamaraju, P. H. Levine

## Abstract

112 Tunisian patients with rapidly progressing breast cancer (RPBC) were entered into a clinical trial evaluating combination chemotherapy as a primary form of treatment before surgery or radiotherapy. Three cycles of cyclophosphamide, methotrexate, and 5-fluorouracil (CMF) were administered at monthly intervals; patients were then randomized to surgery or radiotherapy to control the primary tumour, and 12 more cycles of CMF followed local/regional therapy. RPBC was sensitive to CMF; after only 3 cycles, 11% of evaluable patients showed complete remission and 78% had at least 25% diminution in tumour size. The disease-free interval (DFI) was substantially greater in this series than in a previously reported series treated by surgery and/or radiotherapy alone. No difference in DFI was found between patients randomized to receive surgery and those randomized to receive radiotherapy. Postmenopausal patients responded to CMF as well as premenopausal patients. Combination chemotherapy appears to play an important role in the control of RPBC, an aggressive malignancy often resembling inflammatory breast cancer.


*Br. J. Cancer* (1982) **45**, 367

### PRELIMINARY RESULTS OF PRIMARY SYSTEMIC CHEMOTHERAPY IN ASSOCIATION WITH SURGERY OR RADIOTHERAPY IN RAPIDLY PROGRESSING BREAST CANCER

N. MOURALI*, F. TABBANE*, L. R. MUENZ†, J. BAHI*, S. BELHASSEN*, L. S. KAMARAJU† AND P. H. LEVINE†

*From the *Institut Salah Azaiz, Tunis, Tunisia and the †Division of Cancer Cause and Prevention, National Cancer Institute, Bethesda, Md.* 20205, *U.S.A.*

Received 1 July 1981 Accepted 10 November 1981

**Summary.—112 Tunisian patients with rapidly progressing breast cancer (RPBC) were entered into a clinical trial evaluating combination chemotherapy as a primary form of treatment before surgery or radiotherapy. Three cycles of cyclophosphamide, methotrexate, and 5-fluorouracil (CMF) were administered at monthly intervals; patients were then randomized to surgery or radiotherapy to control the primary tumour, and 12 more cycles of CMF followed local/regional therapy. RPBC was sensitive to CMF; after only 3 cycles, 11% of evaluable patients showed complete remission and 78% had at least 25% diminution in tumour size. The disease-free interval (DFI) was substantially greater in this series than in a previously reported series treated by surgery and/or radiotherapy alone. No difference in DFI was found between patients randomized to receive surgery and those randomized to receive radiotherapy. Postmenopausal patients responded to CMF as well as premenopausal patients. Combination chemotherapy appears to play an important role in the control of RPBC, an aggressive malignancy often resembling inflammatory breast cancer.**

Over the past 10 years we have reported the high frequency of an acute form of breast cancer in Tunisia called rapidly progressing breast cancer (RPBC) or poussée évolutive (PEV). Characterized by rapid growth (PEV 1) and/or objective signs of inflammation (PEV 2 and PEV 3), this form has a brief disease-free interval (DFI) despite vigorous local/regional treatment. Metastases occur frequently and manifest the same aggressiveness as the primary tumour, which explains the poor prognosis, the 5-year survival being under 20% (Mourali *et al.*, 1975, 1977; Tabbane *et al.*, 1977).

Analysis of the natural history suggested that RPBC is mainly a systemic disease at the outset, needing systemic treatment to control the disseminated microfoci, and that local/regional treatment applied to the primary tumour is inadequate for long-term control. Therefore, a new study was initiated using chemotherapy as the major primary treatment modality and, after considering the drugs currently used for breast cancer, a regimen of cyclophosphamide, methotrexate and 5-fluorouracil (CMF) was selected because of its overall effectiveness already reported in advanced breast cancer (Carter, 1980) and its relatively low toxicity. We further evaluated a series of parameters, particularly PEV stage, initial tumour size and menopausal status, to determine whether any of them were able to predict initial response to chemotherapy and/or length of remission. In addition to CMF, our therapeutic trial

Address for reprints: Paul H. Levine, M.D., NCI, NIH, Landow 1D-12, Bethesda, Md. 20205.

included a comparison between radiotherapy and surgery for local/regional treatment.

### MATERIALS AND METHODS

All patients under the age of 65 seen at the Institut Salah Azaiz of Tunis between 1 January 1977 and 30 June 1979 were eligible for this study if their initial visit and subsequent evaluation provided evidence of RPBC without detectable metastases. After physical examination and X-rays of the breast, chest and pelvis, supplemented by other examinations if needed, the patients were staged according to T, N, M, and PEV criteria as previously described (Mourali *et al.*, 1975, 1977; Tabbane *et al.*, 1977). The diagnosis of PEV 1, the mildest form of RPBC, is subjective, determined only by history of rapid tumour growth without inflammatory signs. In PEV 2, the inflammation is limited to less than half the breast, while in PEV 3 inflammation involves more than half the breast.

Once the primary classification was made, patients underwent a surgical biopsy. If the tumour was small, it was frequently removed completely. For larger tumours, a *quartier d'orange*, including skin, was obtained for histological study. The diagnosis was made by frozen section, and when cancer was confirmed, an additional study was systematically carried out to detect occult abdominal metastasis; premenopausal patients were studied at the time of oophorectomy and postmenopausal patients were examined by laparoscopy. All patients aged less than 65 with no evidence of metastasis received an explanation of the protocol of treatment, which included chemotherapy (CMF) and randomization to surgery or radiotherapy. Those accepting the protocol were entered into the study.

The treatment protocol consisted of 3 cycles of CMF, followed by local/regional treatment with surgery or radiotherapy, and then 15 more cycles of CMF. The patients were allocated at random to surgery and radiotherapy in all cases when local/regional conditions permitted; in one patient, radiotherapy was contraindicated because the tumour was extensively ulcerated.

Under the CMF regimen, patients received the following at each cycle: oral cyclophosphamide, 100 mg/m$^2$ every day from Days 1 to 14, i.v. methotrexate, 40 mg/m$^2$ on Days 1 and 8, and i.v. 5-fluorouracil, 600 mg/m$^2$ on Days 1 and 8. Two weeks of rest were observed between Day 14 of one cycle and Day 1 of the next.

WBC and platelet counts were made before the first and eighth day of each cycle. The patients' toxicities were graded from 0 to 2 according to the results of these counts (Bonadonna *et al.*, 1977) to determine the dose of CMF as follows:

- Grade 0 myelosuppression: The calculated dose was given.
- Grade 1 myelosuppression: 50% of the calculated dose was given
- Grade 2 myelosuppression: No chemotherapy was given until the counts returned to at least Grade 1.

Blood was obtained monthly for routine chemical assays of serum creatinine, BUN, calcium, bilirubin, alkaline phosphatase and SGOT.

After 3 cycles, patients without detectable metastases were allocated at random to surgery or radiotherapy for local/regional treatment. Surgery consisted of radical or modified radical mastectomy; radiotherapy was given at a dose of 45 Gy, with subsequent supplementary radiation to the residual tumour and to any adenopathy. Chemotherapy was continued after these local/regional procedures. During the first 3 cycles of CMF, each patient had monthly physical examinations and particular attention was paid to the tumour size; mammography was repeated before randomization.

Tumour response was graded as follows:

- Complete response (CR): disappearance of all clinical and radiological evidence of tumour.
- Partial response (PR): more than 50% reduction in primary tumour size, as measured by the product of the two largest perpendicular diameters of measurable lesions.
- Objective improvement (OI): a decrease in primary tumour size of 25–50%
- No response (NR): $<25\%$ decrease in primary tumour size, or progression in disease.

The results of treatment were first evaluated by the tumour decrease after the first 3 cycles of CMF. Variables considered as prognostic for the initial response to chemotherapy were those with significantly different distributions among the response

TABLE I.—*Distribution of 112 patients with rapidly progressing breast cancer (RPBC) according to their T and N status*

| | | T0 | T1 | T2 | T3 | T4 | TM* | Total |
|---|---|---|---|---|---|---|---|---|
| PEV 1 | N0 | — | — | — | 2 | — | — | 28 |
| | N1 | — | 1 | 10 | 5 | 1 | 2 | |
| | N2 | — | 1 | 1 | 3 | 1 | 1 | |
| | N3 | — | — | — | — | — | — | |
| PEV 2 | N0 | — | — | 2 | 2 | — | — | 39 |
| | N1 | | 1 | 7 | 10 | — | 1 | |
| | N2 | — | — | 1 | 11 | 2 | — | |
| | N3 | — | — | 1 | 1 | — | — | |
| PEV 3 | N0 | — | — | — | 1 | — | — | 45 |
| | N1 | 1 | | 4 | 6 | 1 | 2 | |
| | N2 | — | — | 3 | 13 | 7 | 2 | |
| | N3 | — | — | — | 3 | 2 | — | |
| Total | | 1 | 3 | 29 | 57 | 14 | 8 | 112 |

*TM = multiple primaries.

levels from "complete" to "no response". When the prognostic factor was ordered (*e.g.* T of TNM), we looked for trends in the proportion of responders at each factor level.

In addition to the initial effect of chemotherapy on tumour size, a preliminary evaluation of the long-term effect of chemotherapy as disease control was determined by measuring the interval between the first CMF dose and the first evidence of distant metastases. In this analysis a "score test" (Cox & Hinkley, 1974) was used to see whether the rates of metastasis per patient day (assuming exponentiality) differed between levels of a prognostic factor. Protocol violations were patients who refused further treatment, and these are included in all analyses for the period over which it was possible to follow them. Of the 31 patients who did not complete chemotherapy, 24 had fewer than 5 cycles, and the mean and median number of cycles were 5 and 3 respectively.

### RESULTS

### *Patient population*

Between 1 January 1977 and 30 June 1979, 112 patients entered this study. Sixty-eight were premenopausal and 44 were postmenopausal. The mean age of the patient group was 45 years (range 25–66). Staging by TNM (Table I) revealed a high frequency of large tumours (51% were T3 and 12·5% were T4) and of considerable regional lymph-node involvement (42% were N2); all were MO.

### *Initial response of the primary tumour to chemotherapy*

For the evaluation of the tumour response to the first 3 cycles of CMF, 21 patients could not be used because the entire primary tumour was removed at the diagnostic biopsy. An additional 2 patients were excluded because they did not return for a third measurement. One patient could not be evaluated because she had no measurable tumour; the diagnosis had been made on the basis of a positive biopsy of an axillary lymph node and a positive cytology from the ipsilateral bleeding nipple.

Of the 88 evaluable patients, 10 (11%) showed complete response, 45 patients (51%) had regression >50% (partial response), 14 (16%) had tumour regression of 25–50% (objective improvement), while the remaining 19 (22%) patients did not respond to treatment (Table II).

The initial response to chemotherapy was assessed with respect to the patient's age, the initial pre-treatment tumour size,

TABLE II.—*Relationship between PEV stage and response to chemotherapy* (88 *evaluable patients*)

| Menopausal status | CR | | | PR | | | OI | | | NR | | | Total |
|---|---|---|---|---|---|---|---|---|---|---|---|---|---|
| | Pre- | Post- | Tot. (%) | Pre- | Post- | Tot. (%) | Pre- | Post- | Tot. (%) | Pre- | Post- | Tot. (%) | |
| PEV 1 | 2 | 2 | 4 (25) | 4 | 3 | 7 (44) | 2 | 1 | 3 (19) | 2 | 0 | 2 (12) | 16 |
| PEV 2 | 2 | 0 | 2 (6) | 13 | 8 | 21 (66) | 3 | 0 | 3 (9) | 5 | 1 | 6 (19) | 32 |
| PEV 3 | 3 | 1 | 4 (10) | 10 | 7 | 17 (42) | 2 | 6 | 8 (20) | 5 | 6 | 11 (28) | 40 |
| | | | 10 (11) | | | 45 (51) | | | 14 (16) | | | 19 (22) | 88 |

CR = Complete response.
PR = Partial response (>50%).
OI = Objective improvement (25–50%).
NR = No Response (<25% decrease or increase).

TABLE III.—*Relationship between initial tumour size and response to chemotherapy*

| | No. (%) with CR | No. (%) with PR | No. (%) with OI | No. (%) with NR | Total No. |
|---|---|---|---|---|---|
| T0+T1 | — | — | — | — | 0 |
| T2 | 4 (20) | 12 (60) | 1 (5) | 3 (15) | 20 |
| T3 | 4 (8) | 23 (48) | 9 (19) | 12 (25) | 48 |
| T4 | 1 (8) | 5 (38) | 3 (23) | 4 (31) | 13 |
| TM | 1 (14) | 5 (72) | 1 (14) | 0 (0) | 7 |

the PEV classification, and the menopausal status. The most prominent contributory factor was age, younger patients being more refractory to treatment; 32% of patients under age 40 did not respond to chemotherapy, for example, as compared to 29% of those between 40 and 45, 16% of those between 46 and 50, and only 5% of those over 51. Tumour regression was more pronounced in PEV1 than in PEV3, only 2 of 16 (12%) PEV1 patients failing to respond, compared to 11 of 40 (28%) of PEV3 patients (Table II). Pre-treatment tumour size was also a factor, large tumours demonstrating less decrease than small ones (Table III). Menopausal status did not influence tumour response (Table II).

*Effect of combined treatment (CMF and local/regional treatment) on length of remission*

For the overall response to therapy, all 112 patients were included in the analysis while they complied with the chemotherapy protocol. Forty-seven patients completed all 18 cycles of chemotherapy without developing metastases, 22 developed metastases during the course of chemotherapy, 12 were still under treatment free of disease, and 31 were dropped from the study (30 decided to terminate

FIG. 1.—The correlation between PEV stage and metastasis-free interval; 43 PEV3 patients (—●—) developing metastases earlier than 28 PEV1 (—○—) or 39 PEV2 patients (···▲···.) Entire line represents the total of 112 patients.

treatment and one had to be removed from the trial because of intractable cystitis), though they were retained in the analysis until they left the study. The overall response to therapy (Fig. 1) showed that at 2 years the estimated (life table) proportion of disease-free patients was 59%. The median disease-free interval (31 months) compared favourably with our previous group of patients with PEV (Tabbane *et al.*, 1977) which showed an overall median disease-free interval of 18 months. A comparison of the 2 groups by PEV level revealed an improvement in disease-free interval for each PEV stage,

TABLE IV.—*Comparison of current series with previously reported series**

| PEV | No. of cases | | No. with metastases | | Median metastasis-free (mths) | | Average metastasis-free (mths) | |
|---|---|---|---|---|---|---|---|---|
| 1 | 21* | 28 | 12* | 6 | 23* | >26 | 31·7* | 85 |
| 2 | 29* | 39 | 14* | 11 | 26* | 33 | 30·4* | 68 |
| 3 | 152* | 45 | 73* | 20 | 16* | 19 | 26·6* | 32 |
| Total | 202* | 112 | 99* | 37 | 18* | 31 | 27·8* | 51 |

* Tabbane *et al.*, 1977.

FIG. 2.—90 RPBC patients were treated for local/regional disease with radiotherapy (46 pts, —●—) as compared with those treated with surgery (44 pts, ···○···). There is no significant difference in metastasis-free period between treatment.

FIG. 4.—Relationship between initial tumour size and metastasis-free interval. There is a significant difference between patients with large tumours (T4, 14pts) and those with small tumours (T2, 29 pts) ($P = 0{\cdot}005$, log-rank test). T3 (57 pts) *vs* T4 is non-significant ($P = 0{\cdot}07$).

FIG. 3.—Relationship between menopausal status and metastasis-free interval. No difference was found between premenopausal (68 pts, ···○···) and postmenopausal patients (44 pts, —●—) in their response to treatment.

but the effect appeared to be less prominent in the PEV 3 category (Table IV). However, no significance levels can be attributed to these purely historical comparisons.

Of the 112 patients entered into the study, 90 completed the first 3 cycles of chemotherapy, and were available for random allocation to either surgery or radiotherapy for control of the primary tumour. Of these 90, 2 were not randomized (one refused surgery and received radiotherapy, while the other had extensive ulceration and required surgery). For the whole group, no difference in length of remission was seen between the patients randomized to surgery and those receiving radiotherapy (Fig. 2), but in the PEV3 patients there appeared to be a slightly longer disease-free interval (DFI) in those randomized to radiotherapy than in those receiving survery ($P = 0{\cdot}1$).

Four factors were evaluated for a possible relationship to length of remission: PEV stage; T and N status; initial response to chemotherapy, and menopausal status. Only menopausal status was unrelated to DFI (Fig. 3). A severe PEV grade (Fig. 1) and a large tumour before chemotherapy (Fig. 4) had a strong correlation with early metastasis, while marked lymph-node involvement (N2 or N3) and a poor initial response to chemotherapy had a weaker correlation with early metastasis. This last association was mostly noted in PEV3, where mean DFI (assuming exponential distribution) was: complete regression, 4 years; $>50\%$ regression, 3·1 years; $<50\%$ regression, 2·1 years; stable or larger, 1·7 years.

*Tolerance to chemotherapy*

Mild signs of toxicity under the CMF regimen (alopecia, nausea, vomiting, cystitis) were nearly constant. Almost all the patients received only half-dosages of CMF at least once because of Grade I myelosuppression, and 75% received more than 10% of their cycles in half-dosages; 5% received more than 50% of their cycles in half-doses; 20% had one or several delayed cycles because of Grade II myelosuppression. Two patients could not continue CMF therapy because of severe toxicity—one after 10 cycles (haemorrhagic cystitis) and one before randomization (severe myelotoxicity). Long-term side effects were minimal, however, and no fatality or long-term disability could be attributed to CMF therapy.

### DISCUSSION

It is now widely accepted that adjuvant chemotherapy plays an important role in the treatment of breast cancer. The results of numerous clinical trials have been encouraging and chemotherapy has significantly improved the prognosis of breast cancer. This is due to the effectiveness of multiple drug therapy in the control of disseminated microfoci, and explains the decrease in clinically detectable metastases.

Because of the poor results in the control of RPBC or PEV in our previous series with local/regional treatment associated with surgical oophorectomy in premenopausal women, we initiated this study combining chemotherapy with treatment of the primary lesion to determine whether RPBC is sensitive to chemotherapy and will respond to a combination of systemic and local/regional treatment.

To the best of our knowledge, however, there have been no reports of chemotherapy as a *primary* modality in the treatment of breast cancer, and particularly in RPBC, which includes many cases of inflammatory breast cancer detected in our Institute. Whilst other workers have initiated aggressive chemotherapy protocols for the treatment of inflammatory breast cancer, their results have not yet been published.

Before discussing our results, it is important to note the effect of such a protocol on our patient population. Compliance in a long chemotherapy protocol with randomization of treatment of the primary was difficult for our patients, particularly those from rural areas. Protocol violations were more common in those randomized to surgery than to radiation, for example. It is unlikely, however, that more sophisticated hospital techniques would have detected earlier metastases and affected our patient population, even though 3 patients showed detectable metastases before randomization. In addition to X-ray and bone scan, laparotomy or laparoscopy was used routinely in our series and we frequently detected occult metastases by these procedures, which are not routinely applied by European and American hospitals. The aggressiveness of RPBC has provided us with an excellent indicator of the effectiveness of systemic chemotherapy. We were not only interested in the initial decrease in the size of the primary tumour, but also in prolongation of survival. Of the parameters that appeared to be the best predictors of initial response to chemotherapy, most important were age, tumour size, and PEV stage (PEV with prominent inflammatory signs generally did poorly).

While we found no difference in remission length between PEV1 and PEV2 patients receiving radiation for control of the primary tumour and those receiving surgery, PEV3 patients treated with radiotherapy did slightly better than those treated with surgery.

Previous workers have suggested that both forms of local control involve significant risks, particularly because of their possible depression of the immune system. Primary surgery had been contraindicated in the treatment of PEV at the Institute Gustave-Roussy (France) where the PEV classification originated, because it was considered that surgery would have an immunosuppressive effect and RPBC was thought to be the result of initial patient immunosuppression (Lacour & Hourtoule, 1967). We have shown in our previous studies, however, that RPBC is associated with normal or increased cellular immunity (CMI) to microbial antigens, chemical sensitizing agents, and tumour-related antigens (Mourali *et al.*, 1978; Levine *et al.*, 1981). Stjernsward *et al.* (1976) had earlier warned of the possible risks of radiation-induced immunosuppression in the treatment of breast cancer, but in longitudinal studies on our patients (unpublished), both delayed hypersensitivity and *in vitro* evidence of CMI have remained intact in patients receiving surgery or radiotherapy, and neither regimen appears to offer significant advantages over the other. Our future studies will combine both modalities of treatment according to the areas of clinical involvement.

Another finding of importance was the similar response to our protocol in both pre- and postmenopausal patients. This was significant not only because it emphasized the importance of chemotherapy in postmenopausal patients who did not have oophorectomy at the initial staging, but also because it showed that chemotherapy can be beneficial in postmenopausal patients. Bonnadonna *et al.* (1977) had previously abandoned the CMF regimen in postmenopausal patients because of the poor results in their initial series, but they suggested that the failures in this group might have been due to the relatively short duration (9 cycles) of chemotherapy. Our favourable response with 18 cycles confirms their hypothesis, and prolonged chemotherapy should be considered in other clinical trials in postmenopausal patients.

Although not enough patients have been followed for long enough for us to determine the effect on length of remission and, ultimately, on survival, the relationship between decrease in tumour size and the length of remission, as well as the high frequency of initial response to chemotherapy, suggests to us that chemotherapy will play an important role in many of our patients. As seen in Table III, there already appears to be a significant increase in the median remission length in our patients receiving combination therapy over our initial series.

We are fully aware of the pitfalls in using historical controls, and we cannot make firm conclusions as to the long-term results of our treatment until all patients in the current group have been followed for 5 years (a relatively short follow-up is reasonable in our patients because of the fulminating nature of the disease in the vast majority). Our early results are promising, however, and will form the basis of future trials using other chemotherapeutic agents, an evaluation of the sequencing of treatment regimens, and close scrutiny of the variables that determine the response of our patients to treatment. This latter parameter, which has shown us that patients with PEV3, large tumours and extensive lymph-node involvement are likely to relapse earlier, has already demonstrated the need for more aggressive therapy in these patients. Furthermore, since we know that ~75% of patients respond to chemotherapy, we are evaluating whether an initial debulking procedure such as that described for Burkitt's lymphoma (Magrath *et al.*, 1974) will improve our results, particularly with the routine use of radiotherapy in the treatment of RPBC.

This work was supported by DHEW, NIH Research Project Agreement No. 07002-1. The authors wish to thank Mr Richard Cooper and Ms Marie Topor for their valuable contributions in logistics and data collection.

### REFERENCES

Bonadonna, G., Rossi, A., Valagussa, P., Banfia, A. & Veronesi, U. (1977) CMF program for operable breast cancer with positive axillary nodes: Updated analysis on disease-free interval, site of relapse and drug tolerance. *Cancer*, **39**, 2904.

Carter, S. K. (1980) Surgery plus adjuvant chemotherapy. A review of therapeutic implications. I Breast cancer. *Cancer Chemother. Pharmacol.* **4**, 147.

Cox, D. R. & Hinkley, D. V. (1974) *Theoretical Statistics*. London: Chapman and Hall. p. 315.

Lacour, J. & Hourtoule, F. G. (1967) La place de la chirurgie dans le traitement des formes evolutives du cancer du sein. *Mem. Acad Chirurgie*, **93**, 635.

Levine, P. H. Mourali, N., Tabbane, F. & 4 others (1981) Studies on the role of cellular immunity and genetics in the etiology of reapidly progressing breast cancer in Tunisia. *Int. J. Cancer*, **27**, 611.

Magrath, I. T., Lwanga, S., Carwell, W. & Harrison, N. (1974) Surgical reduction of tumour bulk in management of abdominal Burkitt's lymphoma. *Br. Med. J.*, ii, 308.

MOURALI, N., TABBANE, F., VOGT HOERNER, G., JAZIRI, M., CAMMOUN, M. & BEN ATTIA, R. (1975) Choice of treatment according to the rate of growth. In *Int. Congr. Series No. 353*, Amsterdam: Excerpta Medica, p. 11.

MOURALI, N., TABBANE, F., JAZIRI, M., CAMMOUN, F., BEN ATTIA, R. & BELHASSEN, S. (1977) Fulminating breast cancer. In *Prevention and Detection of Cancer, Part I Vol. 1.* (Ed. Nieburgs). New York: Marcel Dekker. p. 545.

MOURALI, N., LEVINE, P. H., TABBANE, F. & 4 others (1978) Rapidly progressing breast cancer (Poussée évolutive) in Tunisia: Studies on delayed hypersensitivity. *Int. J. Cancer*, **22**, 1.

STJERNSWARD, J., MUENZ, L. R. & VON ESSEN, C. F. (1976) Postoperative radiotherapy and breast cancer. *Lancet*, i, 749.

TABBANE, F., MUENZ, L., JAZIRI, M., CAMMOUN, M., BELHASSEN, S. & MOURALI, N. (1977) Clinical and prognostic features of a rapidly progressing breast cancer in Tunisia. *Cancer*, **40**, 376.